# Psychological distress and associated factors among kidney transplant recipients and living kidney donors during COVID-19

**DOI:** 10.1186/s12882-022-02698-7

**Published:** 2022-02-24

**Authors:** Sobhana Thangaraju, Yeli Wang, Terence Kee, Ping Sing Tee, York Moi Lu, Jing Hua Yong, Quan Yao Ho, Ian Tatt Liew, Fiona Foo, Natelie Kwan, Eleanor Ng, Xia He, Constance Lee, Shannon Baey, Jenny Leong, Judy Tan, Rupesh Madhukar Shirore, Tazeen Hasan Jafar

**Affiliations:** 1 Department of Renal Medicine, Singapore General Hospital, Singapore, Singapore; 2 SingHealth Duke-NUS Transplant Centre, Singapore, Singapore; 3 Program in Health Services and Systems Research, Duke-NUS Medical School, Singapore, Singapore; 4 Duke Global Health Institute, Duke University, Durham, NC USA

**Keywords:** COVID-19, Anxiety, Depression, Psychological distress, Kidney transplant

## Abstract

**Background:**

The coronavirus disease 2019 (COVID-19) pandemic has caused significant psychological distress globally. Our study assessed the prevalence of psychological distress and associated factors during COVID-19 pandemic among kidney transplant recipients and kidney donors.

**Methods:**

A cross-sectional survey of 497 participants (325 recipients and 172 donors) was conducted from 1st May to 30th June 2020 in Singapore. The survey questionnaire assessed knowledge levels of COVID-19, socio-demographic data, health status, psychosocial impact of COVID-19, and precautionary behaviors during the pandemic. Psychological distress was defined as having anxiety, depression, or stress measured by the validated Depression, Anxiety and Stress Scale-21. Linear regression analyses were used to assess factors associated with higher psychological distress.

**Results:**

The prevalence of psychological distress was 14.3% (95% confidence interval: 11.5–17.6%) in the overall population; it was 12.8% (9.79–16.6%) in recipients and 13.4% (9.08–19.6%) in donors with no significant difference (*P* = 0.67). Younger age (21–49 vs. ≥50 years), unmarried status, non-Singapore citizen, worse health conditions, and worrying about physical and mental health were associated with higher psychological distress. Malays (versus Chinese), taking precautionary measures (hand sanitization), and receiving enough information about COVID-19 were associated with lower psychological distress. No interactions were observed between recipients and donors.

**Conclusions:**

At least one in ten recipients and donors suffer from psychological distress during COVID-19 pandemic**.** Focused health education to younger adults, unmarried individuals, non-Singapore citizens, and those with poor health status could potentially prevent psychological distress in recipients and donors.

**Supplementary Information:**

The online version contains supplementary material available at 10.1186/s12882-022-02698-7.

## Background

The Coronavirus disease (COVID-19) outbreak has resulted in significant public health burdens globally; over 150 million confirmed cases and two million deaths have been reported from 223 countries, areas or territories as of 27 February 2021 [1]. Many countries have responded to the outbreak by adopting multi-faceted interventions including lockdowns, travel restrictions, social distancing, and protective measures (e.g., wearing masks) [2]. However, the disruptions in economic development, personal routines, and social interactions may have posed heavy psychological distress worldwide. Systematic reviews and meta-analyses of up to 68 studies from 19 countries found that around 30% of the general population and 55% of high-risk patients (e.g., cancer, type 2 diabetes, COVID-19) had symptoms of anxiety or depression during COVID-19 [3, 4]. By evaluating psychological distress and its associated factors, urgent interventions to mitigate its impact can be initiated during future pandemics.

Female gender, younger adults, lower socioeconomic status, longer media exposure, and individuals having pre-existing physical conditions have been identified by previous studies of the general population as risk factors for increased psychological distress [4]. Patients with chronic kidney disease (CKD), especially those who are on chronic immunosuppression following kidney transplant surgeries, are at particularly high risk of COVID-19 infection and mortality [5–7]. To the best of our knowledge, the prevalence of psychological distress and associated factors during the COVID-19 pandemic have not been explored among kidney transplant recipients and living kidney donors. This knowledge would be especially informative as a recent study showed that stress-related disorders increase the risk of CKD progression and acute kidney injury by 23% [8].

Therefore, we aimed to evaluate the prevalence of, and factors associated with psychological distress during the COVID-19 pandemic among kidney transplant recipients and living kidney donors and compare the differences between the two groups. We hypothesize that the prevalence of psychological distress during the COVID-19 pandemic is significantly higher among kidney transplant recipients compared to donors; and younger age and lack of knowledge regarding COVID-19 will be associated with higher odds of psychological distress.

## Methods

### Study design and study population

We performed a cross-sectional, hospital-based survey on kidney transplant recipients and donors who were on follow-up care with Singapore General Hospital (SGH). The kidney transplant program at SGH has the highest number of recipients and donors under its follow-up in Singapore. The survey was conducted between 1st May to 30th June 2020, which coincided with the legislative nationwide lockdown. Transplant coordinators (TCs) contacted all recipients (*n* = 863) and donors (*n* = 270) in the registry for enrollment. Since face-to-face consent was not allowed during the lockdown, verbal informed consent was obtained via a video call before administering the questionnaire. Information sheet and consent form were subsequently sent to consented participants.

Participants could choose between having a video or phone interview with TCs who had undergone training on administering the instrument; or by filling out a self-administered online questionnaire. Each participant filled out the survey once. Surveys were conducted in either English or Mandarin. All surveys were anonymous, and the confidentiality of the information was ensured. The study was approved by the Ethics Review Committee from the SingHealth Centralised Institutional Review Board (2020/2364). The analysis for the current study was approved by the Institutional Review Board, National University of Singapore (NUS-IRB-2020-160).

### Study outcomes and variables

The survey questionnaire was developed in English and translated into Mandarin. The questionnaire assessed (1) socio-demographic status, (2) health status, (3) impact of COVID-19, (4) coping strategies (5) knowledge levels, (6) precautionary measures, and (7) availability of health information during the pandemic. The English questionnaire is presented in the Additional file 1.

The primary outcome was the psychological distress of COVID-19 in the preceding 4 weeks, which was assessed using the Depression, Anxiety and Stress Scale − 21 Items (DASS-21) – a validated questionnaire comprising of three subscales for depression, anxiety, and stress. Cut-off scores of more than 9, 7 and 14 indicated the presence of depression, anxiety, and stress, respectively. The English version [9] and Chinese version [10] of DASS-21 questionnaire have both been validated in studies evaluating the psychological distress in Singapore and China [10–13]. Other components of the survey, as described below, were translated to Chinese by two independent study team members (HX, YW) who are proficient in the language.

Self-reported demographic characteristics included age (21–49, or > 50 years), gender, ethnicity (Chinese, Malay, Indian or others), marital status (married or others), type of housing as a surrogate for socioeconomic status (government housing [HDB/HUDC], or others), employment status (employed, or unemployed), education levels (primary and lower, or secondary and higher), religion (Buddhist, Christian, or others), and residence status (Singaporean citizen or non-citizen). The vast majority of Singaporeans (78.7%) typically live in government housing (HDB/HUDC) [14] while 16.3% who are likely more affluent live in private housing. In Singapore, 74.3% are Chinese, 13.4% are Malays and 9.4% are Indians [15]; therefore, Indians and Malays were defined as ethnic minorities.

Participants were asked to rate their general health condition (poor or fair, or good, very good or excellent), indicate the frequency of hospital admissions and doctor consultations (never, or once or more), the presence of any general respiratory symptoms in the preceding 14 days, and possible first action after getting sick (self-medicate, or seek help from medical staff). For general health condition, “poor or fair” was the reference group, and for the frequency of hospital admissions and doctor consultations, “never” served as the reference group.

Participants were asked about their worries pertaining to their own health, health of household members, finance, mental health, loneliness and isolation, quality of healthcare received, enough supply of food and medications, as well as their confidence in the Singapore government and healthcare system to control the spread of COVID-19. The response using a 4-item Likert scale (e.g., never, sometimes, most of the time or always), was converted to a numerical score (e.g., never = 0, sometimes = 1, most of the time = 2 and always =4) for further analysis.

Knowledge levels of COVID-19 were assessed by ten questions on infection, prevention, and treatment of COVID-19 (e.g., loss of taste and smell can be a possible sign of COVID-19, etc.). Response options of “true”, “false” and “do not know” were computed to form a knowledge score (true = “1”, false/do not know = “0”). The total score ranged from 0 to 10, with a higher score representing better knowledge of COVID-19.

Questions on precautionary measures assessed how often participants stay at home, or adopt hygiene measures (e.g., sanitizing hands, keeping safe distance, wearing a mask, etc.). Responses were recorded using a 4-item Likert scale. Sources of COVID-19 information, frequency of updates and whether they think the information they received from their healthcare providers was enough were used to assess availability of health information.

### Statistical analysis

The sample sizes were calculated using the eq. N = Zα^2^ × P × (1 − P) / d^2^ where α = 0.05, Zα = 1.96, and d = 0.1. The prevalence of psychological distress was estimated at 20% [12, 13]. To ensure adequate power for analysis, we allowed a response rate as low as 40%; therefore, the calculated sample size for each group was 154, and the total sample size was 308.

Since the prevalence of psychological distress was low, we used linear regression analyses to assess associations between patients’ characteristics and psychological distress to capture more information. Model 1 was the univariate model, and Model 2 included socio-demographic variables. Model 3 consisted of all variables in Model 1, and Model 4 was the fully adjusted parsimonious model using the forward stepwise procedure to select variables with *P* < 0.20 from Model 3. We further kept those variables with *P* < 0.05 in the final model (Model 4). Interactions between patients’ characteristics and patient type (recipients or donors) were examined in interaction terms in Model 2 (for socio-demographic variables) and Model 3 (for the remaining factors). All statistical analyses were conducted using STATA version 14.0 (College Station, TX: StataCorp LP), where two-sided *P* value < 0.05 was considered statistically significant.

## Results

Among 1132 (862 recipients and 270 donors) individuals that were asked to participate in the current study, 497 participants (325 recipients and 172 donors) completed the survey. The overall response rate was 43.9% (95% confidence interval: 41.3–46.8%), and it was higher among donors (63.7% [57.8–69.2%]) compared to recipients (37.7% [34.5–41.0%]; *P* < 0.001). The characteristics of recipients and donors are shown in Table 1 and Additional file 2. Among all participants, 69.4% (*n* = 345) were aged 50 years and above, and 65.4% (*n* = 325) were men. In addition, 78.5% (*n* = 390) were Chinese, 13.3% (*n* = 66) were Malays and 4.43% (*n* = 22) were Indians. A similar proportion of respondents (78.6%) were living in government housing as per national census. Among kidney recipients, 13 recipients (4%) had their kidney transplant surgeries less than one year ago, 72 had transplant surgeries between 1- < 5 years ago, and the remaining recipients (*n* = 240; 73.8%) had their transplant surgeries for 5 years and longer. The prevalence of psychological distress was 14.3% (11.5–17.6%) in the overall study population, and it was 12.8% (9.79–16.6%) in recipients and 13.4 (9.08–19.6%) in donors with no statistically significant difference (*P* = 0.67). The prevalence of its component (depression, anxiety, and stress) was also similar between recipients and donors (all *P*s > 0.05). Similarly, the overall and subscale mean DASS-21 score was comparable between recipients and donors (all *P*s > 0.05).

**Table 1:** Characteristics of the respondents – overall and by participant type (recipient/donor)^a^

Variables	All	Participant type		***P***-value^b^
		Recipients	Donors	
	(***n*** = 497)	(***n*** = 375)	(***n*** = 172)	
Having psychosocial disorders^c^				0.67
No	426 (85.7)	277 (85.2)	149 (86.6)	
Yes	71 (14.3)	48 (14.8)	23 (13.4)	
Having depression^c^				0.23
No	455 (91.6)	294 (90.5)	161 (93.6)	
Yes	42 (8.45)	31 (9.54)	11 (6.40)	
Having anxiety^c^				0.98
No	451 (90.7)	295 (90.8)	156 (90.7)	
Yes	46 (9.26)	30 (9.23)	16 (9.30)	
Having stress^c^				0.93
No	483 (97.2)	316 (97.2)	167 (97.1)	
Yes	14 (2.82)	9 (2.77)	5 (2.91)	
**Demographic variables**				
Age				0.90
21–49	152 (30.8)	100 (30.8)	52 (30.2)	
50 and above	345 (69.4)	225 (69.2)	120 (70.0)	
Gender				**0.012**
Men	325 (65.4)	161 (71.2)	65 (37.8)	
Ethnicity				0.23
Chinese	390 (78.5)	260 (80.0)	130 (75.6)	
Malay	66 (13.3)	36 (11.1)	30 (17.4)	
Indian	22 (4.43)	16 (4.92)	6 (3.49)	
Others	19 (3.82)	13 (4.00)	6 (3.49)	
Marital status				**0.010**
Married	372 (74.9)	231 (71.1)	141 (82.0)	
Others	125 (25.2)	94 (28.9)	31 (18.0)	
Home type				0.30
HDB/HUDC	390 (78.6)	251 (77.2)	139 (81.3)	
Others	106 (21.4)	74 (22.8)	32 (18.7)	
Employment status				0.12
Employed	327 (65.8)	206 (63.4)	121 (70.4)	
Unemployed	170 (34.2)	119 (36.6)	51 (29.7)	
Educational level				0.71
Primary and lower	74 (14.9)	47 (14.5)	27 (15.7)	
Secondary and above	423 (85.1)	278 (85.5)	145 (84.3)	
Religion				0.59
Buddhist	164 (33.0)	109 (33.5)	55 (32.0)	
Christian	114 (22.9)	70 (21.5)	44 (25.6)	
Others	219 (44.1)	146 (44.9)	73 (42.4)	
Resident status in Singapore				0.18
Singapore citizen	472 (95.0)	305 (93.9)	167 (97.1)	
Non-Singapore citizen	25 (5.03)	20 (6.16)	5 (2.91)	

^a^Data are expressed as mean (standard deviation) for continuous variables, and n (percentage) for categorical variables

^b^*P* values were calculated using Student’s t-test for continuous variables, and chi-square test or Fisher’s exact test for categorical variables

^c^Psychosocial disorder was measured using the DASS-21 – a 21-item system that provides independent measures of depression, stress, and anxiety with recommended severity thresholds

Cut-off scores > 9, > 7, and > 14 indicate a positive screen for depression, anxiety, and stress, respectively. Having psychosocial disorders was defined as having depression, anxiety, or stress

The prevalence of psychological distress stratified by participants’ characteristics is shown in Table 2. The prevalence of psychological distress was higher among younger adults (21–49 vs. ≥50 years old), Chinese (versus Malays), unmarried individuals, non-Singapore citizens and those of worse health status or had worries about their physical and mental health or had received insufficient information about COVID-19 situation in Singapore (Table 2).

**Table 2:** Prevalence of psychosocial disorders – overall and by participant type (recipient/donor)^a^

Variables	Prevalence of psychosocial disorders (95% confidence interval)		
	Overall	Kidney recipients	Kidney donors
	(***n*** = 497)	(***n*** = 375)	(***n*** = 172)
**Demographic variables**			
Age			
21–49	21.1% (15.3–28.2%)	22.0% (15.0–21.0%)	19.2% (10.8–31.9%)
50 and above	11.3% (8.38–15.1%)	11.6% (8.01–16.4%)	10.8% (6.44–17.7%)
Gender			
Men	13.7% (9.84–18.8%)	14.3% (9.72–20.5%)	12.3% (6.37–22.5%)
Women	14.8% (11.0–19.5%)	15.2% (10.5–21.5%)	14.0% (8.68–21.9%)
Ethnicity			
Chinese	15.6% (12.4–19.6%)	15.8% (11.8–20.7%)	15.4% (10.2–22.6%)
Malay	6.06% (2.38–14.6%)	5.56% (1.54–18.2%)	6.67% (1.85–21.3%)
Indian	13.6% (4.75–33.3%)	12.5% (3.50–36.0%)	16.7% (3.01–56.4%)
Others	15.8% (5.52–37.6%)	23.1% (8.18–50.3%)	0
Marital status			
Married	12.6% (9.63–16.4%)	12.1% (8.52–17.0%)	13.5% (8.80–20.1%)
Others	19.2% (13.3–27.0%)	21.3% (14.2–30.6%)	12.9% (5.13–28.9%)
Number of people living in home			
1–2 persons	12.3% (7.84–18.8%)	13.0% (7.62–21.4%)	10.9% (4.73–23.0%)
3 or more persons	15.0% (11.7–19.1%)	15.5% (11.4–20.7%)	14.3% (9.23–21.5%)
Home type			
HDB/HUDC	14.1% (11.0–17.9%)	15.1% (11.2–20.1%)	12.2% (7.78–18.7%)
Others	15.0% (9.42–22.9%)	13.5% (7.51–23.1%)	18.2% (8.61–34.4%)
Employment status			
Employed	15.3% (11.8–19.6%)	16.0% (11.6–21.6%)	14.1% (8.96–21.4%)
Unemployed	12.4% (8.22–18.1%)	12.6% (7.79–19.8%)	11.8% (5.50–23.4%)
Educational level			
Primary and lower	5.41% (2.12–13.1%)	2.13% (3.80–11.1%)	11.1% (3.85–28.1%)
Secondary and above	15.8% (12.7–19.6%)	21.3% (14.2–30.6%)	13.8% (9.11–20.3%)
Religion			
Buddhist	15.9% (11.1–22.2%)	16.5% (10.7–24.6%)	14.5% (7.56–26.2%)
Christian	22.8% (16.1–31.3%)	24.3% (15.8–35.5%)	20.5% (11.2–34.5%)
Others	8.68% (5.63–13.2%)	8.90% (5.27–14.6%)	8.22% (3.82–16.8%)
Resident status in Singapore			
Singapore citizen	13.6% (10.8–16.9%)	13.4% (10.1–17.7%)	13.8% (9.35–19.8%)
Non-Singapore citizen	28.0% (14.3–47.6%)	33.3% (16.3–56.3%)	0
**Health status during COVID-19 Pandemic**			
General health condition (self-reported health)			
Poor or fair	27.8% (19.6–37.8%)	29.0% (19.6–40.6%)	23.8% (10.6–45.1%)
Good, very good or excellent	11.3% (8.58–14.7%)	10.9% (7.68–15.4%)	11.9% (7.67–18.1%)
Number of hospital admissions since Feb 2020			
Never	13.4% (10.5–16.9%)	14.1% (10.5–18.8%)	12.1% (7.88–18.1%)
Once or more	19.7% (12.1–30.4%)	17.9% (10.0–29.8%)	26.7% (10.9–52.0%)
Doctor consultations in a clinic or emergency department since Feb 2020			
Never	11.7% (8.67–15.7%)	12.0% (8.40–16.9%)	11.1% (6.32–18.8%)
Once or more	19.1% (13.9–25.6%)	21.0% (14.02–30.0%)	16.4% (9.70–26.6%)
Symptoms reported			
No symptoms	10.7% (8.04–14.2%)	10.5% (7.32–14.9%)	11.2% (6.90–17.6%)
Symptomatic	27.4% (19.8–36.5%)	30.9% (21.2–42.6%)	21.1% (11.1–36.3%)
**COVID-19 impact on other aspects of life**			
Asked to stay at home or be quarantined by the authorities since Feb 2020?^b^			
No	13.4% (10.6–16.8%)	13.6% (10.3–17.9%)	13.0% (8.63–19.0%)
Yes	33.3% (18.0–53.3%)	37.5% (18.5–61.4%)	25.0% (7.15–59.1%)
How likely do you think you would contract COVID-19 during the current outbreak?^b^			
Extremely unlikely	9.46% (4.66–18.3%)	5.00% (1.38–16.5%)	14.7% (6.45–30.1%)
Unlikely	12.7% (9.55–16.7%)	12.3% (8.70–17.1%)	13.6% (8.27–21.5%)
Likely	23.3% (15.1–34.2%)	31.8% (20.0–46.6%)	10.3% (3.58–26.4%)
Extremely likely	57.1% (25.0–84.2%)	75.0% (30.1–95.4%)	33.3% (6.15–79.3%)
Are you worried about the health of your household members during the COVID-19 Pandemic?^b^			
Never	4.76% (2.05–10.7%)	3.85% (13.2–10.7%)	7.41% (2.06–23.4%)
Sometimes	11.5% (8.20–16.0%)	12.6% (8.37–18.5%)	9.68% (5.18–17.4%)
Most of the time	30.1% (20.8–41.4%)	31.1% (19.5–45.7%)	28.6% (15.3–47.1%)
Always	35.0% (15.5–37.7%)	29.4% (16.8–46.2%)	18.2% (7.31–38.5%)
Are you worried that you may not have enough money during the COVID-19 Pandemic?^b^			
Never	9.05% (5.87–13.7%)	7.38% (4.17–12.7%)	13.1% (6.79–23.8%)
Sometimes	16.1% (11.6–21.9%)	17.5% (11.7–25.6%)	13.9% (7.95–23.2%)
Most of the time	16.7% (8.32–30.6%)	22.2% (10.6–40.8%)	6.67% (1.19–29.8%)
Always	28.6% (17.9–42.4%)	32.4% (19.1–49.2%)	20.0% (7.05–45.2%)
Are you worried about your mental health during the COVID-19 Pandemic?^b^			
Never	7.76% (5.42–11.0%)	7.63% (4.88–11.7%)	8.00% (4.40–14.1%)
Sometimes	31.5% (23.6–40.7%)	32.4% (22.9–43.7%)	29.7% (17.5–45.8%)
Most of the time	50.0% (23.7–76.3%)	42.9% (15.8–75.0%)	66.7% (20.8–93.9%)
Always	25.0% (8.89–53.2%)	42.9% (15.8–75.0%)	0
Are you worried that you may feel lonely and isolated during the COVID-19 Pandemic?^b^			
Never	9.16% (6.66–12.5%)	9.64% (6.56–13.9%)	8.27% (4.68–14.2%)
Sometimes	30.5% (22.2–40.4%)	30.2% (20.2–42.4%)	31.3% (18.0–48.6%)
Most of the time	36.4% (15.2–64.6%)	28.6% (8.22–64.1%)	50.0% (15.0–85.0%)
Always	50.0% (18.8–81.2%)	60.0% (23.1–88.2%)	0
Do you agree that the quality of healthcare provided to you have worsened during the COVID-19 Pandemic?^b^			
Extremely disagree	13.3% (8.60–20.1%)	13.4% (8.00–21.6%)	13.2% (5.76–27.3%)
Disagree	14.2% (10.7–18.5%)	14.9% (10.7–20.5%)	12.7% (7.74–20.2%)
Agree	17.1% (8.52–31.3%)	13.6% (4.75–33.3%)	21.1% (8.51–43.3%)
Extremely agree	28.6% (8.22–64.1%)	50.0% (15.0–85.0%)	0
Are you confident that the government and healthcare system of Singapore will be able to control the spread of COVID-19 in Singapore?^b^			
Extremely unconfident	25.0% (7.15–59.1%)	66.7% (20.8–93.9%)	0
Unconfident	37.9% (22.7–56.0%)	41.2% (21.6–64.0%)	22.2% (13.8–60.9%)
Confident	13.8% (10.4–18.1%)	13.5% (9.53–18.9%)	14.3% (8.86–22.2%)
Extremely confident	10.3% (6.37–16.4%)	11.3% (6.45–19.2%)	8.33% (3.29–19.6%)
Are you worried about coming to hospital for your follow-up visits or getting admitted to hospital during the COVID-19 Pandemic?^b^			
Never	7.55% (4.70–11.9%)	8.97% (5.32–14.7%)	4.48% (1.54–12.4%)
Sometimes	14.8% (10.4–20.7%)	14.9% (9.52–22.6%)	14.7% (8.19–25.0%)
Most of the time	31.0% (20.6–43.7%)	28.6% (17.2–43.6%)	37.5% (18.5–61.4%)
Always	23.8% (13.5–38.5%)	26.1% (12.6–46.5%)	21.1% (8.51–43.3%)
Are you worried that Singapore may not have enough supply of food during the COVID-19 Pandemic?^b^			
Never	11.8% (8.81–15.7%)	11.4% (7.90–16.2%)	12.7% (7.74–20.2%)
Sometimes	19.0% (13.4–26.3%)	21.4% (14.1–31.0%)	15.1% (7.85–27.1%)
Most of the time	33.3% (9.68–70.0%)	25.0% (4.56–69.9%)	50.0% (9.45–90.6%)
Always	25.0% (7.15–59.1%)	66.7% (20.8–93.9%)	0
Are you worried that the supply of medications to Singapore may be reduced during the COVID-19 Pandemic?^b^			
Never	11.7% (8.53–15.7%)	12.0% (8.23–17.1%)	11.0% (6.25–18.6%)
Sometimes	17.2% (12.2–23.7%)	17.7% (11.5–26.2%)	16.4% (9.15–27.6%)
Most of the time	37.5% (18.5–61.4%)	36.4% (15.2–64.6%)	40.0% (11.8–76.9%)
Always	16.7% (3.01–56.4%)	50.0% (9.45–90.6%)	0
**Knowledge levels about COVID-19**			
Knowledge score of COVID-19^c^			
< median value (0- < 8)	14.3% (10.9–18.7%)	15.6% (11.1–21.7%)	12.5% (7.84–19.3%)
≥ median value (8–10)	14.2% (9.95–19.9%)	13.7% (9.05–20.2%)	15.9% (7.93–29.4%)
**Precautionary measures taken during COVID-19**			
How often do you try to stay at home?^d^			
Never	–	–	–
Sometimes	0	0	0
Most of the time	14.6% (10.8–19.4%)	14.3% (9.87–20.2%)	15.1% (9.05–24.2%)
Always	13.6% (8.81–20.5%)	14.6% (8.89–23.0%)	11.1% (4.41–25.3%)
I still have to go to work as I work in essential services	18.2% (11.2–28.2%)	19.1% (9.98–33.3%)	17.1% (8.10–32.7%)
How often do you wash your hands after you touch something?^d^			
Never	–	–	–
Sometimes	22.5% (13.0–35.9%)	21.9% (11.0–38.8%)	23.5% (9.56–47.3%)
Most of the time	19.5% (14.7–25.4%)	22.8% (16.4–30.9%)	14.5% (8.47–23.6%)
Always	7.96% (5.09–12.2%)	6.79% (3.83–11.8%)	10.9% (5.40–20.9%)
When you are in a queue, how often do you make sure you keep a distance of at least 1 m from the person in front of you?^d^			
Never	–	–	–
Sometimes	0	0	–
Most of the time	24.7% (16.4–35.4%)	28.9% (17.7–43.4%)	18.8% (8.89–35.3%)
Always	12.5% (9.66–16.1%)	12.4% (8.98–16.8%)	12.9% (8.20–19.7%)
How often do you cover your mouth when you are coughing or sneezing?^d^			
Never	0	0	0
Sometimes	55.6% (26.7–81.1%)	83.3% (43.7–97.0%)	0
Most of the time	17.0% (10.8–25.9%)	15.6% (8.72–26.4%)	20.0% (9.51–37.3%)
Always	12.9% (9.92–16.7%)	12.8% (9.25–17.6%)	13.1% (8.33–20.0%)
How often do you wear a mask when you go out of the house?^d^			
Never	–	–	–
Sometimes	0	0	0
Most of the time	25.0% (10.2–49.5%)	25.0% (8.89–53.2%)	25.0% (4.56–69.9%)
Always	14.2% (11.3–17.7%)	14.3% (10.9–19.7%)	13.9% (9.38–20.2%)
How often do you wash your hands after you cough, sneeze or rub your nose?^d^			
Never	66.7% (20.8–93.9%)	66.7% (20.8–93.9%)	–
Sometimes	20.8% (12.0–33.5%)	24.2% (12.8–41.0%)	15.0% (5.24–36.0%)
Most of the time	12.4% (8.14–18.3%)	12.2% (7.39–19.4%)	12.8% (5.99–25.2%)
Always	13.9% (10.2–18.5%)	13.5% (9.19–19.5%)	14.4% (8.79–22.8%)
When you are eating dishes with others, how often do you make sure there is a clean spoon or fork or chopstick to transfer food from the dish to your plate?^d^			
Never	23.8% (10.6–45.1%)	25.0% (10.2–49.5%)	2.00% (3.62–62.5%)
Sometimes	16.0% (8.34–28.5%)	23.3% (11.8–40.9%)	5.00% (0.89–23.6%)
Most of the time	21.2% (14.4–30.0%)	24.5% (14.9–37.8%)	17.7% (9.57–30.3%)
Always	11.3% (8.23–15.3%)	10.4% (7.00–15.1%)	13.6% (7.98–22.3%)
How often would you wear a mask at home if you are unwell with a cough?^d^			
Never	15.8% (10.2–23.6%)	16.9% (9.72–27.8%)	14.3% (7.10–26.7%)
Sometimes	16.3% (10.1–25.2%)	16.7% (9.32–28.0%)	15.6% (6.87–31.8%)
Most of the time	16.3% (9.95–26.2%)	19.3% (11.1–31.3%)	10.3% (3.58–26.4%)
Always	11.9% (8.08–17.3%)	10.8% (6.65–17.0%)	14.8% (7.70–26.6%)
**Availability of health information**			
How often do you keep yourself updated about the COVID-19 situation in Singapore?^e^			
Never	50.0% (9.45–90.6%)	100% (20.7–100%)	0
Sometimes	15.0% (7.06–29.1%)	13.0% (4.54–32.1%)	17.7% (6.19–41.0%)
Most of the time	20.8% (15.1–28.0%)	22.0% (14.7–31.5%)	19.0% (10.9–30.9%)
Always	10.7% (7.60–14.7%)	10.8% (7.23–15.8%)	10.3% (5.54–18.5%)
Do you think the information you receive about COVID-19 situation in Singapore is enough?^e^			
No	30.8% (19.9–44.3%)	31.4% (18.6–48.0%)	29.4% (13.3–53.1%)
Yes	12.3% (9.55–15.8%)	12.3% (8.99–16.7%)	12.3% (7.94–18.7%)
Do you think your healthcare provider has given enough information to you about how to look after yourself during the COVID-19 Pandemic?^e^			
No	14.1% (9.68–20.2%)	29.4% (13.3–53.1%)	14.4% (8.94–22.4%)
Yes	14.4% (11.0–18.8%)	14.6% (10.8–19.5%)	13.6% (7.03–24.5%)

^a^Data are expressed as prevalence (95% confidence interval). Psychosocial disorder was measured using the DASS-21 – a 21-item system that provides independent measures of depression, stress, and anxiety with recommended severity thresholds. Cutoff scores > 9, > 7, and > 14 indicate a positive screen for depression, anxiety, and stress, respectively. Having psychosocial disorders was defined as having depression, anxiety, or stress

^b^Four missing values

^c^A knowledge score was created assigning the correct answer with a score of 1, and a wrong answer or a “do not know” response with a score of zero. The total knowledge score ranged between 0 to 10, with a higher score representing better knowledge of COVID-19

^d^Twelve missing values for all the variables in this section

^e^Fifteen missing values for all the variables in this section

Factors associated with psychological distress from univariable and multivariable linear regression models are presented in Additional file 3 and Table 3. In the final model (Model 4), younger age versus older age (21–49 vs. ≥50 years; β-coefficient: 1.30 [95% confidence interval: 0.27 to 2.33]), unmarried versus married status (1.09 [0.04 to 2.14]), non-Singapore citizen versus Singapore citizen (5.02 [2.58 to 7.46]), worse self-perceived health (2.14 [0.94 to 3.34]), more frequent doctor consultations (0.98 [0.04 to 1.92]), presence of general or respiratory symptoms (1.23 [0.10 to 2.36]), worrying about physical health of household members (0.84 [0.27 to 1.41]), worrying about mental health (1.56 [0.72 to 2.41]) and feeling lonely or isolated during COVID-19 (2.28 [1.36 to 3.20]) were associated with higher psychological distress. Malays (versus Chinese: − 2.03 [− 3.39 to − 0.68]), taking precautionary measures (hand sanitization: − 0.84 [− 1.48 to − 0.20]), and receiving enough information about COVID-19 (− 1.97 [− 3.41 to − 0.53]) were associated with lower psychological distress (Table 3). No interaction has been observed between factors of psychological distress with patient type (kidney recipients versus donors).

**Table 3:** Linear regression analyses between characteristics of overall population and psychological distress in the final model^a^

	Final model (Model 4^b^)
**Demographic variables**	
Age	
21–49 (*n* = 152)	Ref
50 and above (*n* = 345)	**−1.30 (− 2.33 to − 0.27)**
Gender	
Men (*n* = 325)	Ref
Women (*n* = 172)	0.26 (− 0.64 to 1.17)
Ethnicity	
Chinese (*n* = 390)	Ref
Malay (*n* = 66)	**−2.03 (− 3.39 to − 0.68)**
Indian (*n* = 22)	−0.65 (− 2.84 to 1.53)
Others (*n* = 19)	**−2.85 (−5.52 to − 0.18)**
Marital status	
Married (*n* = 372)	Ref
Others (*n* = 125)	**1.09 (0.04 to 2.14)**
Resident status in Singapore	
Singapore citizen (*n* = 472)	Ref
Non-Singapore citizen (*n* = 25)	**5.02 (2.58 to 7.46)**
**Health status during COVID-19 Pandemic**	
General health condition (self-reported health)	
Poor or fair (*n* = 99)	Ref
Good, very good or excellent (*n* = 407)	**−2.14 (−3.34 to − 0.94)**
Doctor consultations in a clinic or emergency department since Feb 2020	
Never (*n* = 324)	Ref
Once or more (*n* = 173)	**0.98 (0.04 to 1.92)**
Symptoms reported (Y/N)	
No symptoms (*n* = 391)	Ref
Symptomatic (*n* = 106)	**1.23 (0.10 to 2.36)**
**COVID-19 impact on other aspects of life**^**c**^	
Are you worried about the health of your household members during the COVID-19 Pandemic?	**0.84 (0.27 to 1.41)**
Are you worried about your mental health during the COVID-19 Pandemic?	**1.56 (0.72 to 2.41)**
Are you worried that you may feel lonely and isolated during the COVID-19 Pandemic?	**2.28 (1.36 to 3.20)**
**Knowledge levels about COVID-19**	
Knowledge levels about COVID-19	
**Precautionary measures taken during COVID-19**^d^	
How often do you wash your hands after you cough, sneeze or rub your nose?	**−0.84 (−1.48 to − 0.20)**
**Availability of health information**^**e**^	
Do you think the information you receive about COVID-19 situation in Singapore is enough?	
No (*n* = 170)	Ref
Yes (*n* = 312)	**−1.97 (−3.41 to − 0.53)**

^a^Data are expressed as beta-coefficients (95% confidence interval) from linear regression models

^b^The final model (Model 4) was the parsimonious model comprised all factors in model 3 (as shown in Supplemental Table S1) and used forward selection method (*P* < 0.05)

^c^One missing value

^d^Four missing values

^e^Six missing values

To test the robustness of our results, in the sensitivity analysis, we used five age groups instead of the binary age variable. Similar to the current results, younger versus older age (21–39 vs. 50–59 years; 2.44 [0.75 to 4.13]; 21–39 vs. 60–69 years; 2.25 [0.54 to 3.96]) was associated with higher psychological distress.

## Discussion

In the current study in Singapore, we found that at least one in ten kidney transplant recipients and donors suffered from psychological distress during the COVID-19 pandemic. In the overall population, younger age (21–49 vs. ≥50 years), unmarried status, non-Singapore citizenship, worse health conditions, and worrying about physical and mental health were associated with higher psychological distress. Malays (versus Chinese), taking precautionary measures (hand sanitization), and receiving enough information about COVID-19 were associated with lower psychological distress. The relationship of the determinants with psychological distress did not vary between the recipients and donors. Interventions such as targeted-health education including encouragement towards physical exercise, more frequent telehealth consults and rapid access to mental health care or supportive online groups for younger adults, unmarried individuals, non-Singapore citizens, and those with worse health conditions could potentially reduce the risk of psychological distress in these vulnerable groups [16–19].

The prevalence of psychological distress among kidney donors who were largely healthy (13.4 [9.08–19.6%]), was much lower compared to the 30% among a predominantly general population reported from 68 studies in 19 countries during the COVID-19 pandemic [3, 4]. The lower prevalence of psychological distress in Singapore compared to the rest of the world was also observed in a prior study conducted among healthcare workers (7% [95% confidence interval: 5–9%] versus 26% [18–34%]) [3, 13]. Of note, Singapore also had one of the lowest number of COVID-19 cases (10,473 per million population as of 14 February 2021) and mortality (5 deaths per million population) [1] globally, which was likely due to efficient national responses and high-quality medical care. Specifically, since the start of the outbreak, the Singapore government had proactively established official COVID-19 resources and subscription services, as well as frequent briefings and press conferences by the Prime Minister and other officials, to keep the public informed of the latest COVID-19 situation in Singapore [20]. In addition, protective measures such as wearing masks and social distancing were mandated by the law and strictly enforced in Singapore since April 2020 [21], while free masks and sanitizers were distributed across the country. Furthermore, easy access to free COVID-19 testing, robust contact-tracing, and sufficient medical care capacities were instrumental in arresting the spread of COVID-19 and related mortality; various resources and schemes were also promptly established to target financial disruptions, and 24-h National Care hotlines and other online platforms were provided to alleviate the worries of the public [22–25]. In the current study, more than 90% of both recipients and donors showed confidence in the government and healthcare system of Singapore to control the spread of COVID-19, which suggests that the existing comprehensive interventions may have contributed to the low prevalence of psychological distress.

It is also interesting to observe a low prevalence of psychological distress among high-risk kidney transplant recipients in the current study (12.8% [9.79–16.6%]), which was substantially lower compared to the 55% among other high-risk patients (e.g. cancer, type 2 diabetes, COVID-19) [3], and the 39% (clinical anxiety or depression symptoms) among patients with end-stage kidney disease on hemodialysis [26]. In addition to the effective national responses, the low psychological distress among recipients could also be attributed to the high-quality medical care and intensive follow-ups by the SGH transplant program [27]. Since the COVID-19 outbreak, the SGH transplant program has adopted a multi-pronged approach to alleviate the impact of the pandemic including rapid transition to video and tele-consults to minimize potential patient exposure to COVID-19, ensuring safe paths for patients who needed to come to hospital, ensuring a stable supply chain of immunosuppression, and sustaining patient and staff education programs via video conferencing [27]. Specifically, two COVID-19 webinars (on May 9th, 2020 and May 30th, 2020) were held to improve COVID-19 knowledge of recipients, in addition to the official social media platform for the kidney recipients which provided a portal for dissemination of electronic education material and peer-support [28, 29]. As a result of the intensive education, recipients had higher COVID-19 knowledge compared to donors and were more likely to adopt precautionary measures (e.g., hand sanitization), and report that they have received enough COVID-19 information from healthcare providers (79.3% versus 36.2%). Adopting precautionary measures and receiving enough information about COVID-19 were both independently associated with lower psychological distress in the current study, suggesting that the intensive intervention and health education at SGH could have contributed to the substantially lower prevalence of psychological distress among the high-risk kidney transplant recipients in the current study. Nevertheless, we noted that only 36.2% of the donor population thought that they have received enough information from healthcare providers. Programs in Singapore and elsewhere should proactively engage their kidney donors for health education especially during public health crisis.

In addition, the higher psychological distress during COVID-19 among non-Singapore citizens versus Singapore citizens could be partly attributed to outbreaks of COVID-19 occurring in dormitories of migrant workers in Singapore [30, 31]; the potentially higher expenses of medical care for non-Singapore citizens might have also contributed to the higher psychological distress. Thus, proactive health education, access to mental health counselling services, high quality affordable medical care and avenues to alleviate the economic impact of the pandemic must include this vulnerable population to reduce health inequities locally and globally [32–34]. It is also important to underscore that Malay ethnicity was independently associated with lower psychological distress compared to Chinese ethnicity. This was consistent with findings during non-COVID periods in Singapore [35], and might be explained by differences in religious beliefs, strengths of family ties and social networks between the Malay and Chinese communities. Our results were in contrast to studies in Canada, United Kingdom and United States where ethnic minorities had worse mental health [36, 37], and further studies are warranted to understand the unique protective factors of the Malay community in Singapore. Although the prevalence of psychological distress was relatively low among kidney recipients and donors, targeting younger adults, unmarried individuals, non-Singapore citizens, and those with worse health conditions could further improve psychological distress in high-risk kidney transplant recipients and kidney donors.

It is noteworthy that the current study was conducted before the availability of COVID-19 vaccines. As of 10 December 2021, 96% of the Singapore’s eligible population (aged 12 years or older) has been fully vaccinated against COVID-19 [38]. Since the vaccines have significant potential to reduce COVID-19-associated morbidity and mortality among recipients and donors [39, 40] and to improve the mental health distress [41, 42], the high vaccination rate in Singapore and subsequent boosters, as indicated, may have the potential to reduce the psychological distress among recipients and donors as observed in the current study. Further studies are warranted to validate our findings.

To the best of our knowledge, this is the first study assessing psychological distress and associated factors among kidney transplant recipients and donors. We approached all kidney recipients and donors in our registry for study enrollment, and our results could be generalized to countries with similarly low COVID-19 transmission rates. In addition, we used a validated instrument (DASS-21) in assessing psychological distress, thus ensuring the validity of our study outcomes.

However, our study had several limitations. First, potential selection bias may exist as we recruited patients from a single medical center. However, our center is the largest kidney transplant program in Singapore and provides medical care to majority of kidney transplant recipients in Singapore. Second, we observed a low response rate for the current study (43.9% [41.3–46.8%]), which may be due to the lack of face-to-face communication. However, 40% response rate is not unusual for remotely administered anonymous surveys [43, 44]. Moreover, we accounted for a response rate of 40% in calculating the sample size, thus ensuring sufficient sample size and statistical power for the current analysis. Since all surveys were anonymous in the current study, we were not able to compare the characteristics between those we responded to our survey compared to those who did not. Third, the number of patients with psychological distress was relatively small; therefore, the power to demonstrate associations in multivariable adjusted analyses may be limited. Fourth, the present study did not have information on the graft function or immunosuppressive regimens, which may also impact the anxiety levels of transplant patients [45]. Fifth, the current study was a cross-sectional survey, and we could not adjust for baseline prevalence of psychological distress and mental health diagnoses; thus, the observed associations in the current study should be considered correlative not causative. Sixth, our results may not be generalizable to low-income settings where kidney recipients and donors may not receive sufficient medical care or countries with high COVID-19 transmission rates. In addition, some of the variables (e.g., having worries about physical or mental health, and feeling lonely and isolated) may not be entirely independent from symptoms of psychological distress; due to time constraints of the pandemic, we did not develop a pilot survey to standardize or validate all the questions in our study unlike the DASS-21 instrument and hence the results of those questions will require validation in other study populations. However, the primary outcome was based on the DASS-21 scale, which is validated for psychological distress and has been used previously in studies in Singapore [13].

In conclusion, we observed that at least one in ten kidney transplant recipients and donors suffered from psychological distress during COVID-19 pandemic. Younger age, unmarried status, non-Singapore residence, worse health conditions, and worrying about physical and mental health were associated with higher psychological distress. Malays (versus Chinese), taking precautionary measures (hand sanitization), and receiving enough information about COVID-19 were associated with lower psychological distress. Focused health education targeting younger adults, unmarried individuals, non-Singapore citizens, and those with worse health conditions could potentially prevent psychological distress in high-risk kidney transplant recipients and the donor population.

## Supplementary Information

**Additional file 1.** Survey questionnaire - Knowledge, Attitudes and Emotional Responses of Kidney Transplant Recipients during the COVID-19 Pandemic in Singapore. This is the English survey questionnaire used in the current study.

**Additional file 2: Supplemental Table S1**. Characteristics of the respondents – overall and by participant type (recipient/donor). The table shows the characteristics of the respondents and by participant type.

**Additional file 3: Supplemental Table S2**. Linear regression analyses between characteristics of overall population and psychological distress in univariable and multivariable models. The table shows the univariable and multivariable models between characteristics of overall population and psychological distress.

## Data Availability

The data and material are available based on reasonable requests from corresponding authors and approval by IRB (contact person: Ms. Sapna Menon; email: sapna.menon@duke-nus.edu.sg).

## Abbreviations

CKD: Chronic kidney disease
SGH: Singapore General Hospital
TC: Transplant coordinators
DASS-21: Depression, Anxiety and Stress Scale − 21 Items
