# Supplementary material for: Psychological distress and associated factors among kidney transplant recipients and living kidney donors during COVID-19
Source: BMC Nephrol. 2022 Feb 24;23:80. doi: 10.1186/s12882-022-02698-7 (PMC8867454; doi:10.1186/s12882-022-02698-7)
Supplement: Supplementary file 1 — Additional file 1. Survey questionnaire - Knowledge, Attitudes and Emotional Responses of Kidney Transplant Recipients during the COVID-19 Pandemic in Singapore. This is the English survey questionnaire used in the current study. [file 12882_2022_2698_MOESM1_ESM.docx]

**Additional file 1**

**Survey questionnaire - Knowledge, Attitudes and Emotional Responses of Kidney Transplant Recipients during the COVID-19 Pandemic in Singapore**

**Section 1. Demographic variables**

**This section is to gather some information about yourself**

1. **Please indicate who are you? (please pick one)**

Kidney Transplant Recipient Living Kidney Donor

1. **What is your gender? (please tick one)**

Male Female

1. **What is your age? (please tick one)**

21- 29 30- 39 40- 49 50- 59 60- 69 70 and above

1. **What is your race? (please tick one)**

Chinese Malay Indian Eurasian Others

1. **What is your marital status? (please tick one)**

Single Married Divorced or Separated Widowed

1. **How many people are living in your home? (please tick one)**

1 person 2 persons 3-5 persons 6 persons or more

1. **What is the type of home you stay in? (please tick one or more)**

Rented/serviced apartment HDB/HUDC Private apartment Landed property

1. **What is your employment status? (please tick one)**

Unemployed Retired Student Employed

**Please indicate your specific occupation if employed**: ____________________

1. **What is your educational level? (please tick one)**

None Primary Secondary Diploma Degree and above

1. **What is your religion? (please tick one or more)**

Buddhist Christian Hindu Muslim Free-thinker Other religions

1. **How often do you stay with a healthcare provider or someone working in the hospital or clinic? (please tick one)**

Never Sometimes Most of the time Always

1. **How long ago was your transplant? (please tick one if you are a kidney transplant recipient, do not tick if you are a kidney donor)**

Less than 1 year 1 to <5 years 5 to <10 years

10 to <15 years 15 to <20 years 20 years or more

1. **What type was your kidney donor? (please tick one if you are a kidney transplant recipient, do not tick if you are a kidney donor)**

Deceased donor in Singapore Living donor in Singapore Overseas donor

1. **What is your resident status in Singapore? (please tick one)**

Singapore citizen Singapore permanent resident Non-resident

**Section 2. Health status during COVID-19 Pandemic**

**This section is to determine your general health during this period.**

1. **With respect to your general health condition now, how do you feel about your general health now? (please tick one)**

Poor Fair Good Very good Excellent

1. **How often have you been admitted to hospital since Feb 2020? (please tick one)**

Never Once Twice >2 times

1. **How often have you consulted a doctor in a GP clinic or Polyclinic clinic or Emergency Department so far since Feb 2020? (please tick one)**

Never Once Twice >2 times

1. **Do you have any of the following symptoms in the last 14 days? (please tick one or more)**

Fever Cough Sore throat Running nose Diarrhea

Shortness of breath Tiredness Muscle pain Headache

Sputum production Loss of taste and/or smell None of the above

1. **If you fall sick, what would be the first thing you would do? (please tick one or more)**

Self-medicate and see if I can get better first

Call the transplant coordinator

See my nearby general practitioner

See a doctor in the polyclinic

Go to emergency department of a hospital

Arrange an appointment to see my kidney specialist in the hospital

***Section 3. Impact of COVID-19**

**This section used the DASS-21 Depression Anxiety Stress Scale Test to evaluate the severity of depression, anxiety and stress associated with COVID-19.**

**Please describe how often do you have the following feelings regarding COVID-19 over the past 4 weeks (please tick one):**

1. **I found it hard to wind down.**

Did not apply to me at all

Applied to me to some degree, or some of the time

Applied to me to a considerable degree or a good part of time

Applied to me very much or most of the time

1. **I was aware of dryness of my mouth.**

Did not apply to me at all

Applied to me to some degree, or some of the time

Applied to me to a considerable degree or a good part of time

Applied to me very much or most of the time

1. **I couldn’t seem to experience any positive feeling at all.**

Did not apply to me at all

Applied to me to some degree, or some of the time

Applied to me to a considerable degree or a good part of time

Applied to me very much or most of the time

1. **I experienced breathing difficulty (e.g. excessively rapid breathing, breathlessness in the absence of physical exertion).**

Did not apply to me at all

Applied to me to some degree, or some of the time

Applied to me to a considerable degree or a good part of time

Applied to me very much or most of the time

1. **I found it difficult to work up the initiative to do things.**

Did not apply to me at all

Applied to me to some degree, or some of the time

Applied to me to a considerable degree or a good part of time

Applied to me very much or most of the time

1. **I tended to over-react to situations.**

Did not apply to me at all

Applied to me to some degree, or some of the time

Applied to me to a considerable degree or a good part of time

Applied to me very much or most of the time

1. **I experienced trembling (e.g. in the hands).**

Did not apply to me at all

Applied to me to some degree, or some of the time

Applied to me to a considerable degree or a good part of time

Applied to me very much or most of the time

1. **I felt that I was using a lot of nervous energy.**

Did not apply to me at all

Applied to me to some degree, or some of the time

Applied to me to a considerable degree or a good part of time

Applied to me very much or most of the time

1. **I was worried about situations in which I might panic and make a fool of myself.**

Did not apply to me at all

Applied to me to some degree, or some of the time

Applied to me to a considerable degree or a good part of time

Applied to me very much or most of the time

1. **I felt that I had nothing to look forward to.**

Did not apply to me at all

Applied to me to some degree, or some of the time

Applied to me to a considerable degree or a good part of time

Applied to me very much or most of the time

1. **I found myself getting agitated.**

Did not apply to me at all

Applied to me to some degree, or some of the time

Applied to me to a considerable degree or a good part of time

Applied to me very much or most of the time

1. **I found it difficult to relax.**

Did not apply to me at all

Applied to me to some degree, or some of the time

Applied to me to a considerable degree or a good part of time

Applied to me very much or most of the time

1. **I felt down-hearted and blue.**

Did not apply to me at all

Applied to me to some degree, or some of the time

Applied to me to a considerable degree or a good part of time

Applied to me very much or most of the time

1. **I was intolerant of anything that kept me from getting on with what I was doing.**

Did not apply to me at all

Applied to me to some degree, or some of the time

Applied to me to a considerable degree or a good part of time

Applied to me very much or most of the time

1. **I felt I was close to panic.**

Did not apply to me at all

Applied to me to some degree, or some of the time

Applied to me to a considerable degree or a good part of time

Applied to me very much or most of the time

1. **I was unable to become enthusiastic about anything.**

Did not apply to me at all

Applied to me to some degree, or some of the time

Applied to me to a considerable degree or a good part of time

Applied to me very much or most of the time

1. **I felt I wasn’t worth much as a person.**

Did not apply to me at all

Applied to me to some degree, or some of the time

Applied to me to a considerable degree or a good part of time

Applied to me very much or most of the time

1. **I felt that I was rather touchy.**

Did not apply to me at all

Applied to me to some degree, or some of the time

Applied to me to a considerable degree or a good part of time

Applied to me very much or most of the time

1. **I was aware of the action of my heart in the absence of physical exertion (e.g. sense of heart rate increase, heart missing a beat)**

Did not apply to me at all

Applied to me to some degree, or some of the time

Applied to me to a considerable degree or a good part of time

Applied to me very much or most of the time

1. **I felt scared without any good reason.**

Did not apply to me at all

Applied to me to some degree, or some of the time

Applied to me to a considerable degree or a good part of time

Applied to me very much or most of the time

1. **I felt that life was meaningless.**

Did not apply to me at all

Applied to me to some degree, or some of the time

Applied to me to a considerable degree or a good part of time

Applied to me very much or most of the time

**Questions below ask about how COVID-19 has impacted on other aspects of your life so far.**

1. **Have you been asked to stay at home or be quarantined by the authorities since Feb 2020? (please tick one)**

Yes No

1. **Have you been tested for COVID-19? (please tick one)**

Yes No

**If yes, what was your result? (please tick one)**

Positive Negative

1. **How likely do you think you would contract COVID-19 during the current outbreak? (please tick one)**

Extremely unlikely Unlikely Likely Extremely likely

1. **Are you worried about the health of your household members during the COVID-19 Pandemic? (please tick one)**

Never Sometimes Most of the time Always

1. **Are you worried that you may not have enough money during the COVID-19 Pandemic? (please tick one)**

Never Sometimes Most of the time Always

1. **Are you worried about your mental health during the COVID-19 Pandemic ?**

Never Sometimes Most of the time Always

1. **Are you worried that you may feel lonely and isolated during the COVID-19 Pandemic ?**

Never Sometimes Most of the time Always

1. **Do you agree that the quality of healthcare provided to you have worsened during the COVID-19 Pandemic? (please tick one)**

Extremely disagree Disagree Agree Extremely agree

1. **Are you confident that the government and healthcare system of Singapore will be able to control the spread of COVID-19 in Singapore? (please tick one)**

Extremely unconfident Unconfident Confident Extremely confident

1. **Are you worried about coming to hospital for your follow-up visits or getting admitted to hospital during the COVID-19 Pandemic? (please tick one)**

Never Sometimes Most of the time Always

1. **Are you worried that Singapore may not have enough supply of food during the COVID-19 Pandemic? (please tick one)**

Never Sometimes Most of the time Always

1. **Are you worried that the supply of medications to Singapore may be reduced during the COVID-19 Pandemic? (please tick one)**

Never Sometimes Most of the time Always

**Section 4. Coping strategies about your worries of COVID-19**

**This section asks what you have done or anticipate to alleviate your worries:**

1. **What have you done to reduce your worries? (please tick one or more)**

Assessed self-help resources through media or online platforms

Consulted professionals (e.g. psychologists, psychiatrists)

Engage in my hobbies

Rested more or exercised often

Talked with family or friends

1. **What kind of support or services would you expect? (please tick one or more)**

More information via media or online platforms

Help from community services

More support from family and friends

More instructions and information from the transplant team

A patient support group where I can interact with other patients in the same situation

I do not need support

**Section 5. Knowledge levels about COVID-19**

**This section tests your knowledge levels about COVID-19**

1. **Some blood pressure medications should be stopped as they may increase the risk for COVID-19 infections. (please tick one)**

True False Don’t know

1. **Loss of taste and smell can be a possible sign of COVID-19. (please tick one)**

True False Don’t know

1. **Only old people or people with medical conditions can get infected with COVID-19. (please tick one)**

True False Don’t know

1. **It is not possible to get COVID-19 from an infected person who is feeling well. (please tick one)**

True False Don’t know

1. **It is possible to get infected with COVID-19 if you touch your face after holding a door handle used by a person infected with COVID-19. (please tick one)**

True False Don’t know

1. **It is not necessary to wear a mask if you are well. (please tick one)**

True False Don’t know

1. **Hand sanitizer is better than soap and water to wash your hands. (please tick one)**

True False Don’t know

1. **There is a cure for COVID-19. (please tick one)**

True False Don’t know

1. **COVID-19 infection in kidney transplant recipients may be more severe than in other type of patients. (please tick one)**

True False Don’t know

1. **COVID-19 infection can cause permanent injury to the lungs. (please tick one)**

True False Don’t know

**Section 6. Precautionary measures taken during COVID-19**

**This section checks if you are taking precautionary measures during COVID-19 Pandemic**

1. **How often do you try to stay at home? (please tick one)**

Never Sometimes Most of the time Always

I still have to go to work as I work in essential services

1. **How often do you wash your hands after you touch something? (please tick one)**

Never Sometimes Most of the time Always

1. **When you are in a queue, how often do you make sure you keep a distance of at least 1 meter from the person in front of you? (please tick one)**

Never Sometimes Most of the time Always

1. **How often do you cover your mouth when you are coughing or sneezing? (please tick one)**

Never Sometimes Most of the time Always

1. **How often do you wear a mask when you go out of the house? (please tick one)**

Never Sometimes Most of the time Always

1. **How often do you wash your hands after you cough, sneeze or rub your nose? (please tick one)**

Never Sometimes Most of the time Always

1. **When you are eating dishes with others, how often do you make sure there is a clean spoon or fork or chopstick to transfer food from the dish to your plate? (please tick one)**

Never Sometimes Most of the time Always

1. **How often would you wear a mask at home if you are unwell with a cough? (please tick one)**

Never Sometimes Most of the time Always

**Section 7. Availability of health information**

**This section checks if you are receiving enough health information about COVID-19 in Singapore.**

1. **How often do you keep yourself updated about the COVID-19 situation in Singapore? (please tick one)**

Never Sometimes Most of the time Always

1. **Where do you get your information about COVID-19 situation in Singapore? (please tick one or more)**

TV Newspaper Social Media Family members

Friends Chat groups

1. **Do you think the information you receive about COVID-19 situation in Singapore is enough? (please tick one)**

Yes No

1. **Which healthcare provider has provided you information about COVID-19? (please tick one or more)**

No one gives me information My doctor My nurse

My transplant coordinator Other healthcare providers

1. **Do you think your healthcare provider has given enough information to you about how to look after yourself during the COVID-19 Pandemic? (please tick one)**

Yes No

*Questions 20 to 40 in Section 3 are from DASS 21 (https://maic.qld.gov.au/wp-content/uploads/2016/07/DASS-21.pdf).
