# Supplementary material for: Psychological distress and associated factors among kidney transplant recipients and living kidney donors during COVID-19
Source: BMC Nephrol. 2022 Feb 24;23:80. doi: 10.1186/s12882-022-02698-7 (PMC8867454; doi:10.1186/s12882-022-02698-7)
Supplement: Supplementary file 2 — Additional file 2: Supplemental Table S1. Characteristics of the respondents – overall and by participant type (recipient/donor). The table shows the characteristics of the respondents and by participant type. [file 12882_2022_2698_MOESM2_ESM.docx]

**Additional file 2:**

**Supplemental Table S1. Characteristics of the respondents – overall and by participant type (recipient/donor)^a^**

| **Variables** | **All** | **Participant type** | | ***P*-value^b^** |
| --- | --- | --- | --- | --- |
|  |  | **Recipients** | **Donors** |  |
|  | **(*n*=497)** | **(*n*=375)** | **(*n*=172)** |  |
| **Health status during COVID-19 Pandemic** | |  |  |  |
| General health condition (self-reported health) |  |  |  | **0.013** |
| Poor or fair | 99 (18.1) | 69 (21.2) | 21 (12.2) |  |
| Good, very good or excellent | 407 (81.9) | 256 (78.8) | 151 (87.8) |  |
| Number of hospital admissions since Feb 2020 |  |  |  | **0.010** |
| Never | 426 (85.71) | 269 (82.77) | 157 (91.28) |  |
| Once or more | 71 (14.29) | 56 (17.23) | 15 (8.72) |  |
| Doctor consultations in a clinic or emergency department since Feb 2020 |  |  |  | **0.010** |
| Never | 324 (65.19) | 225 (69.23) | 99 (57.56) |  |
| Once or more | 173 (34.81) | 100 (30.77) | 73 (42.44) |  |
| Symptoms reported in the last 14 days |  |  |  | 0.76 |
| No symptoms | 391 (78.67) | 257 (79.08) | 134 (77.91) |  |
| Symptomatic | 106 (21.33) | 68 (20.92) | 38 (22.09) |  |
| Symptoms |  |  |  |  |
| Fever (Y/N) | 14 (2.82) | 8 (2.46) | 6 (3.49) | 0.57 |
| Cough | 18 (3.62) | 13 (4) | 5 (2.91) | 0.53 |
| Sore throat | 16 (3.22) | 8 (2.46) | 8 (4.65) | 0.18 |
| Running nose | 11 (2.21) | 6 (1.85) | 5 (2.91) | 0.52 |
| Diarrhea | 9 (1.81) | 7 (2.15) | 2 (1.16) | 0.72 |
| Shortness of breath | 8 (1.61) | 5 (1.54) | 3 (1.74) | 0.99 |
| Tiredness | 37 (7.44) | 26 (8) | 11 (6.4) | 0.51 |
| Muscle pain | 33 (6.64) | 20 (6.15) | 13 (7.56) | 0.54 |
| Headache | 27 (5.43) | 16 (4.92) | 11 (6.4) | 0.49 |
| Sputum production | 9 (1.81) | 8 (2.46) | 1 (0.58) | 0.17 |
| Loss of taste and/or smell | 1 (0.2) | 1 (0.31) | 0 (0) | 0.99 |
| None of the above | 393 (79.07) | 258 (79.38) | 135 (78.49) | 0.81 |
| Possible first action after getting sick (self-reported) |  |  |  |  |
| Self-medicate and see if I can get better first | 177 (35.61) | 79 (24.31) | 98 (56.98) | **<0.001** |
| Call the transplant coordinator | 88 (17.71) | 85 (26.15) | 3 (1.74) | **<0.001** |
| See my nearby general practitioner | 236 (47.48) | 167 (51.38) | 69 (40.12) | **0.017** |
| See a doctor in the polyclinic | 88 (17.71) | 53 (16.31) | 35 (20.35) | 0.26 |
| Go to emergency department of a hospital | 48 (9.66) | 48 (14.77) | 0 (0) | **<0.001** |
| Arrange an appointment to see my kidney specialist in the hospital | 14 (2.82) | 13 (4) | 1 (0.58) | **0.042** |
| **COVID-19 impact on other aspects of life^c^** | |  |  |  |
| Asked to stay at home or be quarantined by the authorities since Feb 2020^d^ |  |  |  | 0.91 |
| No | 470 (95.14) | 308 (95.06) | 162 (95.29) |  |
| Yes | 24 (4.86) | 16 (4.94) | 8 (4.71) |  |
| Tested for COVID-19 |  |  |  | 0.99 |
| No | 462 (93.52) | 303 (93.52) | 159 (93.53) |  |
| Yes | 32 (6.48) | 21 (6.48) | 11 (6.47) |  |
| COVID-19 test result |  |  |  |  |
| Negative | 32 (100) | 21 (100) | 11 (100) |  |
| How likely do you think you would contract COVID-19 during the current outbreak?^c^ |  |  |  | **0.040** |
| Extremely unlikely | 74 (15.01) | 40 (12.35) | 34 (20.12) |  |
| Unlikely | 339 (68.76) | 236 (72.84) | 103 (60.95) |  |
| Likely | 73 (14.81) | 44 (13.58) | 29 (17.16) |  |
| Extremely likely | 7 (1.42) | 4 (1.23) | 3 (1.78) |  |
| Are you worried about the health of your household members during the COVID-19 Pandemic?^d^ |  |  |  | 0.19 |
| Never | 105 (21.26) | 78 (24.07) | 27 (15.88) |  |
| Sometimes | 260 (52.63) | 167 (51.54) | 93 (54.71) |  |
| Most of the time | 73 (14.78) | 45 (13.89) | 28 (16.47) |  |
| Always | 56 (11.34) | 34 (10.49) | 22 (12.94) |  |
| Are you worried that you may not have enough money during the COVID-19 Pandemic?^d^ |  |  |  | 0.08 |
| Never | 210 (42.51) | 149 (45.99) | 61 (35.88) |  |
| Sometimes | 193 (39.07) | 114 (35.19) | 79 (46.47) |  |
| Most of the time | 42 (8.5) | 27 (8.33) | 15 (8.82) |  |
| Always | 49 (9.92) | 34 (10.49) | 15 (8.82) |  |
| Are you worried about your mental health during the COVID-19 Pandemic?^d^ |  |  |  | 0.93 |
| Never | 361 (73.08) | 236 (72.84) | 125 (73.53) |  |
| Sometimes | 111 (22.47) | 74 (22.84) | 37 (21.76) |  |
| Most of the time | 10 (2.02) | 7 (2.16) | 3 (1.76) |  |
| Always | 12 (2.43) | 7 (2.16) | 5 (2.94) |  |
| Are you worried that you may feel lonely and isolated during the COVID-19 Pandemic?^d^ |  |  |  | 0.91 |
| Never | 382 (77.33) | 249 (76.85) | 133 (78.24) |  |
| Sometimes | 95 (19.23) | 63 (19.44) | 32 (18.82) |  |
| Most of the time | 11 (2.23) | 7 (2.16) | 4 (2.35) |  |
| Always | 6 (1.21) | 5 (1.54) | 1 (0.59) |  |
| Do you agree that the quality of healthcare provided to you have worsened during the COVID-19 Pandemic?^d^ |  |  |  | 0.14 |
| Extremely disagree | 135 (27.33) | 97 (29.94) | 38 (22.35) |  |
| Disagree | 311 (62.96) | 201 (62.04) | 110 (64.71) |  |
| Agree | 41 (8.3) | 22 (6.79) | 19 (11.18) |  |
| Extremely agree | 7 (1.42) | 4 (1.23) | 3 (1.76) |  |
| Are you confident that the government and healthcare system of Singapore will be able to control the spread of COVID-19 in Singapore?^d^ |  |  |  | 0.31 |
| Extremely unconfident | 8 (1.62) | 3 (0.93) | 5 (2.94) |  |
| Unconfident | 29 (5.87) | 17 (5.25) | 12 (7.06) |  |
| Confident | 312 (63.16) | 207 (63.89) | 105 (61.76) |  |
| Extremely confident | 145 (29.35) | 97 (29.94) | 48 (28.24) |  |
| Are you worried about coming to hospital for your follow-up visits or getting admitted to hospital during the COVID-19 Pandemic?^d^ |  |  |  | 0.19 |
| Never | 212 (42.91) | 145 (44.75) | 67 (39.41) |  |
| Sometimes | 182 (36.84) | 114 (35.19) | 68 (40) |  |
| Most of the time | 58 (11.74) | 42 (12.96) | 16 (9.41) |  |
| Always | 42 (8.5) | 23 (7.1) | 19 (11.18) |  |
| Are you worried that Singapore may not have enough supply of food during the COVID-19 Pandemic?^d^ |  |  |  | 0.27 |
| Never | 338 (68.42) | 228 (70.37) | 110 (64.71) |  |
| Sometimes | 142 (28.74) | 89 (27.47) | 53 (31.18) |  |
| Most of the time | 6 (1.21) | 4 (1.23) | 2 (1.18) |  |
| Always | 8 (1.62) | 3 (0.93) | 5 (2.94) |  |
| Are you worried that the supply of medications to Singapore may be reduced during the COVID-19 Pandemic?^d^ |  |  |  | 0.26 |
| Never | 309 (62.55) | 209 (64.51) | 100 (58.82) |  |
| Sometimes | 163 (33) | 102 (31.48) | 61 (35.88) |  |
| Most of the time | 16 (3.24) | 11 (3.4) | 5 (2.94) |  |
| Always | 6 (1.21) | 2 (0.62) | 4 (2.35) |  |
| **Coping strategies about your worries of COVID-19** | | | | |
| What have you done to reduce your worries? |  |  |  |  |
| 1. Assessed self-help resources through media or online platforms | 122 (24.55) | 75 (23.08) | 47 (27.33) | 0.64 |
| 2. Consulted professionals (e.g. psychologists, psychiatrists) | 12 (2.41) | 9 (2.77) | 3 (1.74) | 0.39 |
| 3. Engage in my hobbies | 245 (49.3) | 170 (52.31) | 75 (43.6) | **0.040** |
| 4. Rested more or exercised often | 295 (59.36) | 203 (62.46) | 92 (53.49) | 0.82 |
| 5. Talked with family or friends | 280 (56.34) | 190 (58.46) | 90 (52.33) | 0.61 |
| What kind of support or services would you expect? |  |  |  |  |
| 1.More information via media or online platforms | 208 (41.85) | 132 (40.62) | 76 (44.19) | 0.44 |
| 2.Help from community services | 71 (14.29) | 43 (13.23) | 28 (16.28) | 0.36 |
| 3.More support from family and friends | 193 (38.83) | 136 (41.85) | 57 (33.14) | 0.06 |
| 4.More instructions and information from the transplant team | 164 (33) | 140 (43.08) | 24 (13.95) | **<0.001** |
| 5.A patient support group where I can interact with other patients in the same situation | 46 (9.26) | 42 (12.92) | 4 (2.33) | **<0.001** |
| 6.I do not need support | 117 (23.54) | 69 (21.23) | 48 (27.91) | 0.10 |
| **Knowledge levels about COVID-19** | | | | |
| Some blood pressure medications should be stopped as they may increase the risk for COVID-19 infections. |  |  |  | **0.010** |
| True | 27 (5.57) | 16 (4.98) | 11 (6.71) |  |
| False | 303 (62.47) | 216 (67.29) | 87 (53.05) |  |
| Don't know | 155 (31.96) | 89 (27.73) | 66 (40.24) |  |
| Loss of taste and smell can be a possible sign of COVID-19. |  |  |  | 0.61 |
| True | 368 (75.88) | 248 (77.26) | 120 (73.17) |  |
| False | 53 (10.93) | 33 (10.28) | 20 (12.2) |  |
| Don't know | 64 (13.2) | 40 (12.46) | 24 (14.63) |  |
| Only old people or people with medical conditions can get infected with COVID-19. |  |  |  | 0.26 |
| True | 68 (14.02) | 46 (14.33) | 22 (13.41) |  |
| False | 402 (82.89) | 268 (83.49) | 134 (81.71) |  |
| Don't know | 15 (3.09) | 7 (2.18) | 8 (4.88) |  |
| It is not possible to get COVID-19 from an infected person who is feeling well. |  |  |  | **0.042** |
| True | 55 (11.34) | 30 (9.35) | 25 (15.24) |  |
| False | 394 (81.24) | 271 (84.42) | 123 (75) |  |
| Don't know | 36 (7.42) | 20 (6.23) | 16 (9.76) |  |
| It is possible to get infected with COVID-19 if you touch your face after holding a door handle used by a person infected with COVID-19. |  |  |  | **0.004** |
| True | 447 (92.16) | 305 (95.02) | 142 (86.59) |  |
| False | 18 (3.71) | 7 (2.18) | 11 (6.71) |  |
| Don't know | 20 (4.12) | 9 (2.8) | 11 (6.71) |  |
| It is not necessary to wear a mask if you are well. |  |  |  | 0.21 |
| True | 26 (5.36) | 16 (4.98) | 10 (6.1) |  |
| False | 453 (93.4) | 303 (94.39) | 150 (91.46) |  |
| Don't know | 6 (1.24) | 2 (0.62) | 4 (2.44) |  |
| Hand sanitizer is better than soap and water to wash your hands. |  |  |  | 0.59 |
| True | 84 (17.32) | 58 (18.07) | 26 (15.85) |  |
| False | 353 (72.78) | 229 (71.34) | 124 (75.61) |  |
| Don't know | 48 (9.9) | 34 (10.59) | 14 (8.54) |  |
| There is a cure for COVID-19. |  |  |  | **<0.001** |
| True | 79 (16.29) | 43 (13.4) | 36 (21.95) |  |
| False | 306 (63.09) | 223 (69.47) | 83 (50.61) |  |
| Don't know | 100 (20.62) | 55 (17.13) | 45 (27.44) |  |
| COVID-19 infection in kidney transplant recipients may be more severe than in other type of patients. |  |  |  | **<0.001** |
| True | 385 (79.38) | 285 (88.79) | 100 (60.98) |  |
| False | 27 (5.57) | 9 (2.8) | 18 (10.98) |  |
| Don't know | 73 (15.05) | 27 (8.41) | 46 (28.05) |  |
| COVID-19 infection can cause permanent injury to the lungs. |  |  |  | **0.003** |
| True | 330 (68.04) | 235 (73.21) | 95 (57.93) |  |
| False | 46 (9.48) | 24 (7.48) | 22 (13.41) |  |
| Don't know | 109 (22.47) | 62 (19.31) | 47 (28.66) |  |
| Total knowledge score^e^ | 7.53 (2.23) | 7.94 (1.90) | 6.73 (2.59) | **<0.001** |
| **Precautionary measures taken during COVID-19** | | | | |
| How often do you try to stay at home?^f^ |  |  |  | **0.039** |
| Never | 0 (0) | 0 (0) | 0 (0) |  |
| Sometimes | 15 (3.09) | 8 (2.49) | 7 (4.27) |  |
| Most of the time | 261 (53.81) | 175 (54.52) | 86 (52.44) |  |
| Always | 132 (27.22) | 96 (29.91) | 36 (21.95) |  |
| I still have to go to work as I work in essential services | 77 (15.88) | 42 (13.08) | 35 (21.34) |  |
| How often do you wash your hands after you touch something?^f^ |  |  |  | **0.047** |
| Never | 0 (0) | 0 (0) | 0 (0) |  |
| Sometimes | 49 (10.1) | 32 (9.97) | 17 (10.37) |  |
| Most of the time | 210 (43.3) | 127 (39.56) | 83 (50.61) |  |
| Always | 226 (46.6) | 162 (50.47) | 64 (39.02) |  |
| When you are in a queue, how often do you make sure you keep a distance of at least 1 meter from the person in front of you?^f^ |  |  |  | 0.20 |
| Never | 0 (0) | 0 (0) | 0 (0) |  |
| Sometimes | 1 (0.21) | 1 (0.31) | 0 (0) |  |
| Most of the time | 77 (15.88) | 45 (14.02) | 32 (19.51) |  |
| Always | 407 (83.92) | 275 (85.67) | 132 (80.49) |  |
| How often do you cover your mouth when you are coughing or sneezing?^f^ |  |  |  | 0.97 |
| Never | 3 (0.62) | 2 (0.62) | 1 (0.61) |  |
| Sometimes | 9 (1.86) | 6 (1.87) | 3 (1.83) |  |
| Most of the time | 94 (19.38) | 64 (19.94) | 30 (18.29) |  |
| Always | 379 (78.14) | 249 (77.57) | 130 (79.27) |  |
| How often do you wear a mask when you go out of the house?^f^ |  |  |  | 0.60 |
| Never | 0 (0) | 0 (0) | 0 (0) |  |
| Sometimes | 4 (0.82) | 2 (0.62) | 2 (1.22) |  |
| Most of the time | 16 (3.3) | 12 (3.74) | 4 (2.44) |  |
| Always | 465 (95.88) | 307 (95.64) | 158 (96.34) |  |
| How often do you wash your hands after you cough, sneeze or rub your nose?^f^ |  |  |  | 0.25 |
| Never | 3 (0.62) | 3 (0.93) | 0 (0) |  |
| Sometimes | 53 (10.93) | 33 (10.28) | 20 (12.2) |  |
| Most of the time | 162 (33.4) | 115 (35.83) | 47 (28.66) |  |
| Always | 267 (55.05) | 170 (52.96) | 97 (59.15) |  |
| When you are eating dishes with others, how often do you make sure there is a clean spoon or fork or chopstick to transfer food from the dish to your plate?^f^ |  |  |  | **0.001** |
| Never | 21 (4.33) | 16 (4.98) | 5 (3.05) |  |
| Sometimes | 50 (10.31) | 30 (9.35) | 20 (12.2) |  |
| Most of the time | 104 (21.44) | 53 (16.51) | 51 (31.1) |  |
| Always | 310 (63.92) | 222 (69.16) | 88 (53.66) |  |
| How often would you wear a mask at home if you are unwell with a cough?^f^ |  |  |  | 0.06 |
| Never | 114 (23.51) | 65 (20.25) | 49 (29.88) |  |
| Sometimes | 92 (18.97) | 60 (18.69) | 32 (19.51) |  |
| Most of the time | 86 (17.73) | 57 (17.76) | 29 (17.68) |  |
| Always | 193 (39.79) | 139 (43.3) | 54 (32.93) |  |
| **Availability of health information** | | | | |
| How often do you keep yourself updated about the COVID-19 situation in Singapore?^g^ |  |  |  | 0.10 |
| Never | 2 (0.41) | 1 (0.31) | 1 (0.61) |  |
| Sometimes | 40 (8.3) | 23 (7.21) | 17 (10.43) |  |
| Most of the time | 149 (30.91) | 91 (28.53) | 58 (35.58) |  |
| Always | 291 (60.37) | 204 (63.95) | 87 (53.37) |  |
| Where do you get your information about COVID-19 situation in Singapore?^g^ |  |  |  |  |
| TV | 362 (72.84) | 239 (73.54) | 123 (71.51) | 0.63 |
| Newspaper | 231 (46.48) | 156 (48) | 75 (43.6) | 0.35 |
| Social media | 331 (66.6) | 219 (67.38) | 112 (65.12) | 0.61 |
| Family members | 175 (35.21) | 110 (33.85) | 65 (37.79) | 0.38 |
| Friends | 141 (28.37) | 93 (28.62) | 48 (27.91) | 0.87 |
| Chat groups | 177 (35.61) | 120 (36.92) | 57 (33.14) | 0.40 |
| Do you think the information you receive about COVID-19 situation in Singapore is enough?^g^ |  |  |  | 0.86 |
| No | 52 (10.79) | 35 (10.97) | 17 (10.43) |  |
| Yes | 430 (89.21) | 284 (89.03) | 146 (89.57) |  |
| Which healthcare provider has provided you information about COVID-19?^g^ |  |  |  |  |
| No one gives me information | 194 (39.03) | 75 (23.08) | 119 (69.19) | **<0.001** |
| My doctor | 155 (31.19) | 149 (45.85) | 6 (3.49) | **<0.001** |
| My nurse | 34 (6.84) | 32 (9.85) | 2 (1.16) | **<0.001** |
| My transplant coordinator | 206 (41.45) | 194 (59.69) | 12 (6.98) | **<0.001** |
| Other healthcare providers | 75 (15.09) | 44 (13.54) | 31 (18.02) | 0.18 |
| Do you think your healthcare provider has given enough information to you about how to look after yourself during the COVID-19 Pandemic?^g^ |  |  |  | **<0.001** |
| No | 170 (35.27) | 66 (20.69) | 104 (63.8) |  |
| Yes | 312 (64.73) | 253 (79.31) | 59 (36.2) |  |

^a^Data are expressed as mean (standard deviation) for continuous variables, and n (percentage) for categorical variables.

^b^*P* values were calculated using Student’s t-test for continuous variables, and chi-square test or Fisher’s exact test for categorical variables.

^c^Four missing values.

^d^Three missing values.

^e^A knowledge score was created assigning the correct answer with a score of 1, and a wrong answer or a “do not know” response with a score of zero. The total knowledge score ranged between 0 to 10, with a higher score representing better knowledge of COVID-19.

^f^Twelve missing values for all the variables in this section.

^g^Fifteen missing values for all the variables in this section.
