# Supplementary material for: Psychological distress and associated factors among kidney transplant recipients and living kidney donors during COVID-19
Source: BMC Nephrol. 2022 Feb 24;23:80. doi: 10.1186/s12882-022-02698-7 (PMC8867454; doi:10.1186/s12882-022-02698-7)
Supplement: Supplementary file 3 — Additional file 3: Supplemental Table S2. Linear regression analyses between characteristics of overall population and psychological distress in univariable and multivariable models. The table shows the univariable and multivariable models between characteristics of overall population and psychological distress. [file 12882_2022_2698_MOESM3_ESM.docx]

**Additional file 3:**

**Supplemental Table S2. Linear regression analyses between characteristics of overall population and psychological distress in univariable and multivariable models^a^**

|  |  | **Model 1^b^** | **Model 2^c^** | **Model 3^d^** |  |  |  |  |  |  |
| --- | --- | --- | --- | --- | --- | --- | --- | --- | --- | --- |
| **Demographic variables** |  |  |  |  |  |  |  |  |  |  |
| Age |  |  |  |  |  |  |  |  |  |  |
| 21 – 49 (*n*=152) |  | Ref | Ref | Ref |  |  |  |  |  |  |
| 50 and above (*n*=345) |  | -3.10 (-4.23 to -1.98) | -2.60 (-3.81 to -1.39) | -0.96 (-2.07 to 0.15) |  |  |  |  |  |  |
| Gender |  |  |  |  |  |  |  |  |  |  |
| Men (*n*=325) |  | Ref | Ref | Ref |  |  |  |  |  |  |
| Women (*n*=172) |  | 0.41 (-0.66 to 1.48) | 0.20 (-0.85 to 1.26) | 0.29 (-0.66 to 1.25) |  |  |  |  |  |  |
| Ethnicity |  |  |  |  |  |  |  |  |  |  |
| Chinese (*n*=390) |  | Ref | Ref | Ref |  |  |  |  |  |  |
| Malay (*n*=66) |  | -1.48 (-3.06 to 0.09) | -0.58 (-2.33 to 1.17) | -1.54 (-3.15 to 0.07) |  |  |  |  |  |  |
| Indian (*n*=22) |  | 0.08 (-2.51 to 2.67) | 0.72 (-1.90 to 3.34) | 0.25 (-2.14 to 2.64) |  |  |  |  |  |  |
| Others (*n*=19) |  | 1.97 (-0.81 to 4.75) | -1.30 (-4.39 to 1.80) | -2.70 (-5.50 to 0.10) |  |  |  |  |  |  |
| Marital status |  |  |  |  |  |  |  |  |  |  |
| Married (*n*=372) |  | Ref | Ref | Ref |  |  |  |  |  |  |
| Others (*n*=125) |  | 1.81 (0.60 to 3.03) | 1.59 (0.36 to 2.81) | 1.12 (0.05 to 2.19) |  |  |  |  |  |  |
| Home type |  |  |  |  |  |  |  |  |  |  |
| HDB/HUDC (*n*=390) |  | Ref | Ref | Ref |  |  |  |  |  |  |
| Others (*n*=106) |  | -0.26 (-1.56 to 1.03) | -0.82 (-2.11 to 0.47) | -0.06 (-1.20 to 1.09) |  |  |  |  |  |  |
| Employment status |  |  |  |  |  |  |  |  |  |  |
| Employed (*n*=327) |  | Ref | Ref | Ref |  |  |  |  |  |  |
| Unemployed (*n*=170) |  | -0.66 (-1.78 to 0.46) | 0.32 (-0.82 to 1.46) | -0.25 (-1.28 to 0.79) |  |  |  |  |  |  |
| Educational level |  |  |  |  |  |  |  |  |  |  |
| Primary and lower (*n*=74) |  | Ref | Ref | Ref |  |  |  |  |  |  |
| Secondary and above (*n*=423) |  | 2.11 (0.63 to 3.60) | 0.99 (-0.54 to 2.52) | 1.12 (-0.28 to 2.53) |  |  |  |  |  |  |
| Religion |  |  |  |  |  |  |  |  |  |  |
| Buddhist (*n*=164) |  | Ref | Ref | Ref |  |  |  |  |  |  |
| Christian (*n*=114) |  | 0.94 (-0.50 to 2.38) | 0.93 (-0.52 to 2.37) | 0.95 (-0.31 to 2.21) |  |  |  |  |  |  |
| Others (*n*=219) |  | -0.81 (-2.02 to 0.41) | -0.83 (-2.17 to 0.50) | -0.26 (-1.45 to 0.93) |  |  |  |  |  |  |
| Resident status in Singapore |  |  |  |  |  |  |  |  |  |  |
| Singapore citizen (*n*=472) |  | Ref | Ref | Ref |  |  |  |  |  |  |
| Non-Singapore citizen (*n*=25) |  | 4.92 (2.53 to 7.32) | 4.15 (1.44 to 6.85) | 4.52 (2.03 to 7.02) |  |  |  |  |  |  |
| **Health status during COVID-19 Pandemic** | | |  |  |  |  |  |  |  |  |
| General health condition (self-reported health) |  |  |  |  |  |  |  |  |  |  |
| Poor or fair (*n*=99) |  | Ref |  | Ref |  |  |  |  |  |  |
| Good, very good or excellent (*n*=407) |  | -3.19 (-4.54 to -1.83) |  | -1.94 (-3.19 to -0.68) |  |  |  |  |  |  |
| Number of hospital admissions since Feb 2020 |  |  |  |  |  |  |  |  |  |  |
| Never (*n*=426) |  | Ref |  | Ref |  |  |  |  |  |  |
| Once or more (*n*=71) |  | 1.63 (0.12 to 3.15) |  | 1.03 (-0.31 to 2.37) |  |  |  |  |  |  |
| Doctor consultations in a clinic or emergency department since Feb 2020 |  |  |  |  |  |  |  |  |  |  |
| Never (*n*=324) |  | Ref |  | Ref |  |  |  |  |  |  |
| Once or more (*n*=173) |  | 1.59 (0.48 to 2.70) |  | 0.41 (-0.61 to 1.43) |  |  |  |  |  |  |
| Symptoms reported (Y/N) |  |  |  |  |  |  |  |  |  |  |
| No symptoms (*n*=391) |  | Ref |  | Ref |  |  |  |  |  |  |
| Symptomatic (*n*=106) |  | 2.77 (1.47 to 4.05) |  | 1.10 (-0.05 to 2.26) |  |  |  |  |  |  |
| **COVID-19 impact on other aspects of life^e^** | | |  |  |  |  |  |  |  |  |
| Asked to stay at home or be quarantined by the authorities since Feb 2020 |  |  |  |  |  |  |  |  |  |  |
| No (*n*=470) |  | Ref |  | Ref |  |  |  |  |  |  |
| Yes (*n*=24) |  | 3.76 (1.30 to 6.23) |  | 1.58 (-0.54 to 3.69) |  |  |  |  |  |  |
| How likely do you think you would contract COVID-19 during the current outbreak? |  | 1.91 (1.03 to 2.80) |  | 0.28 (-0.53 to 1.09) |  |  |  |  |  |  |
| Are you worried about the health of your household members during the COVID-19 Pandemic? |  | 1.60 (1.01 to 2.18) |  | 0.63 (-0.02 to 1.28) |  |  |  |  |  |  |
| Are you worried that you may not have enough money during the COVID-19 Pandemic? |  | 1.58 (1.03 to 2.13) |  | 0.22 (-0.34 to 0.79) |  |  |  |  |  |  |
| Are you worried about your mental health during the COVID-19 Pandemic? |  | 3.75 (2.98 to 4.52) |  | 1.62 (0.71 to 2.53) |  |  |  |  |  |  |
| Are you worried that you may feel lonely and isolated during the COVID-19 Pandemic? |  | 4.11 (3.23 to 4.99) |  | 2.11 (1.14 to 3.08) |  |  |  |  |  |  |
| Are you confident that the government and healthcare system of Singapore will be able to control the spread of COVID-19 in Singapore? |  | -1.46 (-2.33 to -0.60) |  | -0.61 (-1.37 to 0.16) |  |  |  |  |  |  |
| Are you worried about coming to hospital for your follow-up visits or getting admitted to hospital during the COVID-19 Pandemic? |  | 1.10 (0.53 to 1.67) |  | -0.01 (-0.57 to 0.55) |  |  |  |  |  |  |
| Are you worried that Singapore may not have enough supply of food during the COVID-19 Pandemic? |  | 1.55 (0.66 to 2.45) |  | -0.69 (-1.64 to 0.26) |  |  |  |  |  |  |
| Are you worried that the supply of medications to Singapore may be reduced during the COVID-19 Pandemic? |  | 1.97 (1.12 to 2.64) |  | 0.30 (-0.67 to 1.27) |  |  |  |  |  |  |
| **Knowledge levels about COVID-19** | | | | | |  |  |  |  |  |
| Knowledge levels about COVID-19 |  | -0.07 (-0.31 to 0.17) |  | -0.04 (-0.30 to 0.23) |  |  |  |  |  |  |
| **Precautionary measures taken during COVID-19**^f^ | | | | | |  |  |  |  |  |
| How often do you wash your hands after you touch something? |  | -1.47 (-2.28 to -0.66) |  | -0.11 (-0.89 to 0.68) |  |  |  |  |  |  |
| When you are in a queue, how often do you make sure you keep a distance of at least 1 meter from the person in front of you? |  | -2.52 (-3.94 to -1.10) |  | -0.35 (-1.74 to 1.04) |  |  |  |  |  |  |
| How often do you cover your mouth when you are coughing or sneezing? |  | -1.66 (-2.70 to -0.61) |  | -0.63 (-1.63 to 0.37) |  |  |  |  |  |  |
| How often do you wash your hands after you cough, sneeze or rub your nose? |  | -0.91 (-1.67 to -0.15) |  | -0.60 (-1.34 to 0.13) |  |  |  |  |  |  |
| When you are eating dishes with others, how often do you make sure there is a clean spoon or fork or chopstick to transfer food from the dish to your plate? |  | -1.10 (-1.73 to -0.47) |  | -0.30 (-0.89 to 0.28) |  |  |  |  |  |  |
| **Availability of health information^g^** | | | | | |  |  |  |  |  |
| How often do you keep yourself updated about the COVID-19 situation in Singapore? |  | -1.14 (-1.92 to -0.35) |  | 0.02 (-0.72 to 0.76) |  |  |  |  |  |  |
| Do you think the information you receive about COVID-19 situation in Singapore is enough? |  |  |  |  |  |  |  |  |  |  |
| No (*n*=170) |  | Ref |  | Ref |  |  |  |  |  |  |
| Yes (*n*=312) |  | -3.50 (-5.16 to -1.83) |  | -1.66 (-3.14 to -0.18) |  |  |  |  |  |  |

^a^Data are expressed as beta-coefficients (95% confidence interval) from linear regression models.

^b^Model 1 was the univariate model.

^c^Model 2 adjusted for demographic and socio-economic variables.

^d^Model 3 included demographic and socio-economic variables, health status during COVID-19 pandemic, COVID-19 impact on life, knowledge levels about COVID-19, precautionary measures taken during COVID-19, and availability of health information during COVID-19.

since Feb 2020, symptoms reported, worrying about health of household during COVID, worrying about mental health and being lonely during COVID, washing hands after cough, sneeze or rub nose, whether having received enough information from healthcare providers.

^e^One missing value.

^f^Four missing values.

^g^Six missing values.
